# Combination of dasatinib and gemcitabine reduces the ALDH1A1 expression and the proliferation of gemcitabine-resistant pancreatic cancer MIA PaCa-2 cells

**DOI:** 10.3892/ijo.2014.2357

**Published:** 2014-03-21

**Authors:** HONG-QUAN DUONG, YONG WEON YI, HYO JIN KANG, INSOO BAE, YOUNG-JOO JANG, SAHNG-JUNE KWAK, YEON-SUN SEONG

**Affiliations:** 1 Department of Nanobiomedical Science and BK21 PLUS Research Center for Regenerative Medicine, Dankook University, Cheonan 330-714, Republic of Korea;; 2 Center of Research and Development, Duy Tan University, K7/25 Quang Trung, Danang, Vietnam;; 3 Department of Biochemistry, College of Medicine, Dankook University, Cheonan 330-714, Republic of Korea;; 4 Departments of Oncology Lombardi Comprehensive Cancer Center, Georgetown University, Washington DC 20057, USA; 5 Radiation Medicine, Lombardi Comprehensive Cancer Center, Georgetown University, Washington DC 20057, USA

**Keywords:** ALDH1A1, gemcitabine, dasatinib, resistance, pancreatic cancer cells, MIA PaCa-2

## Abstract

Gemcitabine-based chemotherapy is the standard for treatment of pancreatic cancer; however, intrinsic and acquired resistance to gemcitabine commonly occurs. Aldehyde dehydrogenase 1A1 (ALDH1A1), one of the characteristic features of tumor-initiating and/or cancer stem cell (CSC) properties, is important in both intrinsic and acquired resistance to gemcitabine. In this study, we investigated the effectiveness of dasatinib, an SRC inhibitor, and gemcitabine combination to inhibit the survivals of parental (MIA PaCa-2/P) and gemcitabine-resistant (MIA PaCa-2/GR) cell lines. In MIA PaCa-2/GR cells, the levels of phospho-SRC and ALDH1A1 were increased compared to MIA PaCa-2/P cells. Inhibition of SRC by dasatinib or siRNA synergistically enhanced gemcitabine-induced anti-proliferative effects and induced apoptotic cell death in these cells. Furthermore, combination of SRC inhibition (either by dasatinib or siRNA) and gemcitabine significantly decreased the levels of ALDH1A1 expression. These results suggest that dasatinib and gemcitabine combination may be a potential therapeutic strategy to overcome gemcitabine resistance by decreasing the levels of ALDH1A1 expression.

## Introduction

With a 5-year survival rate of 5%, pancreatic cancer is one of the most lethal malignancies in the United States (1,2). The poor prognosis of pancreatic cancer is due to the failure of early diagnosis for curative surgical resection combined with adjuvant chemotherapy, the aggressive biological behavior of the tumor and insensitivity to most conventional therapies including chemotherapy and radiotherapy (3). Although a recent study shows that FOLFIRINOX (combination of fluorouracil, leucovorin, irinotecan and oxaliplatin) is superior option for patients with advanced pancreatic cancer and good ECOG (Eastern Cooperative Oncology Group) performance status, gemcitabine (difluorodeoxycitidine, dFdC) still remains the first-line standard drug as a treatment available for metastatic and non-metastatic with locally advanced pancreatic cancer (4,5); however, the disease rapidly develops resistance to the drug and no apparent improvement in overall survival rate over the last decade (2,6).

The aldehyde dehydrogenase (ALDH) family comprises evolutionarily conserved 19 isoforms that catalyze oxidization of aldehydes to carboxylic acids and are localized in the cytoplasm, mitochondria or nucleus of cells (7). Several lines of evidence suggest that ALDH activity is a hallmark of cancer stem cells (CSCs) that can be measured by Aldeflour assay (7). Since CSCs are known to resistant to various chemotherapies, it is possible that the resistance to chemotherapeutic agents is due to the activity of ALDH in CSCs. For example, the expression of ALDH1A1 or ALDH3A1 is correlated with the resistance of chemotherapeutics in hematopoietic stem cells, breast cancer patient samples, or colorectal cancer xenograft tumors (8–10). The activation of ALDH1A1 was found in stem cell population in multiple types of cancers including breast, colon, bladder, pancreatic, head and neck squamous, lung, liver and ovarian cancer (11–18). In pancreatic cancer patients, ALDH1A1 expression is also implicated in a stem cell marker and correlates with worse outcome (19). Recently, we found that the high level of ALDH1A1 expression is correlated with the resistance to gemcitabine in MIA PaCa-2/GR comparing with parental MIA PaCa-2/P cells (20).

Dasatinib is a potent inhibitor of SRC family kinases and ABL kinases (21,22). An increasing body of evidence from preclinical and clinical studies suggests that dasatinib enhanced the antitumor efficacy of various chemotherapeutic drugs in multiple types of cancers such as lung, colon, pancreatic, breast, prostate, ovarian, melanoma and leukemia (23–33). In addition, dasatinib enhanced antitumor effect of etoposide associated with a decreased proportion of aldehyde dehydrogenase 1 (ALDH1)-positive cells in breast cancer MDA-MB-231 and MDA-MB-157 cells (34). The combination treatment of dasatinib and curcumin also eliminated chemoresistant colon cancer HCT-116 and HT-29 cell line which show increased expression of CSC markers as CD133, CD44, CD166 and ALDH1 (35). Although the role of dasatinib in sensitizing pancreatic cancer cells to gemcitabine was explored (25,26), the potentially crucial role in the treatment of pancreatic CSCs has not been investigated.

Herein, we demonstrated the combination effect of dasatinib and gemcitabine in inhibition of the cell proliferation and survival of MIA PaCa-2/P and MIA PaCa-2/GR cell lines that are highly enriched with the level of ALDH1A1 expression. Our results suggest that the synergistic antitumor effects of dasatinib/gemcitabine combination are mediated by reducing the level of ALDH1A1 in ALDH1A1-enriched pancreatic cancer MIA PaCa-2 cells.

## Materials and methods

### Cell culture and reagents

MIA PaCa-2 cells were purchased from American Type Culture Collection (ATCC, Manassas, VA, USA). MIA PaCa-2/GR cells were generated in our laboratory as described (20). The cells were maintained as described previously (20). Gemcitabine and dasatinib were purchased from LC Labs (Woburn, MA, USA) and dissolved in PBS and dimethyl sulfoxide (DMSO), respectively.

### 3-(4,5-dimethylthiazol-2-yl)-2,5-diphenyltetrazolium bromide (MTT) assay

A total of 1,500 cells per well were plated in 96-well flat bottom plates and then exposed to test combination treatment of gemcitabine and dasatinib in various concentrations. The MTT cell viability assay was performed as described previously (20). The combination index (CI) was calculated by using CompuSyn software (ComboSyn, Inc., Paramus, NJ, USA) and values of CI<1, CI=1, CI>1 indicate synergism, additive effect, and antagonism, respectively.

### Clonogenic assay

MIA PaCa-2/P and/or MIA PaCa-2/GR cells (2×10^5^ cells) were seeded in 60-mm dishes. Twenty-four hours after plating, varying concentrations of the drugs, either as a single agent or in combination, were added to the dishes. After treatment, cells (500 cells) were re-seeded in 60 mm dishes in triplicate. After 14 days of incubation, the colonies were stained and examined as described elsewhere (36,37).

### Western blot analysis

Cells were grown to ∼70% confluence and reagents were added as indicated. Western blot analysis was performed as described previously (36,37) with the following antibodies: phospho-SRC (Y416), phospho-STAT3 (T705), and SRC (Cell Signaling Technology, Inc., Boston, MA, USA), ALDH1A1 (Abcam, Cambridge, UK), Poly-ADP-ribose-polymerase (PARP; BD Biosciences, Franklin, NJ, USA), α-tubulin and β-actin (Sigma, St. Louis, MO, USA).

### Caspase-3/7 activity assay

Caspase-3/7 activity assay was carried out by using a Caspase-3/7 Glo assay (Promega, Madison, WI, USA) according to the manufacturer’s protocol. Cells were treated with either gemcitabine, dasatinib alone, or combination of both drugs for indicated concentrations and caspase-3/7 activity was measured from cell lysates after indicated time of treatment. Luminescence was measured at 490 nm using VICTOR X multilable plate readers (Perkin-Elmer Life Sciences, Boston, MA, USA). Relative luminescence units were determined by calculating luminescence values from samples as a percentage of values from vehicle-treated control samples. The experiments were performed in triplicate and repeated on two separately initiated cultures.

### Small interfering RNA (siRNA)

For the RNA interfering experiment, SRC-siRNA, 5’-GUGUCUUAAUACUGUC CUU-3’ and control-siRNA, 5’-GACGAGCGGCACGUG CACA-3’ were purchased from Bioneer (Daejeon, Korea). SRC-siRNA or control-siRNA were transfected into MIA PaCa-2/P and MIA PaCa-2/GR cells using Lipofectamine™ 2000 (Invitrogen, Carlsbad, CA, USA) according to the manufacturer’s procedure. The transfected cells were then processed for western blot analysis, caspase-3/7 activity and MTT cell proliferation assay (20).

### Statistical methods

Statistical comparisons were made using the two-tailed Student’s t-test where appropriate. Results were considered significant in all experiments at ^*^P<0.05, ^**^P<0.01 and ^***^P<0.005. Data were expressed as the mean ± SD.

## Results

### The level of SRC is significantly higher in MIA PaCa-2/GR cells

**Figure 1.**
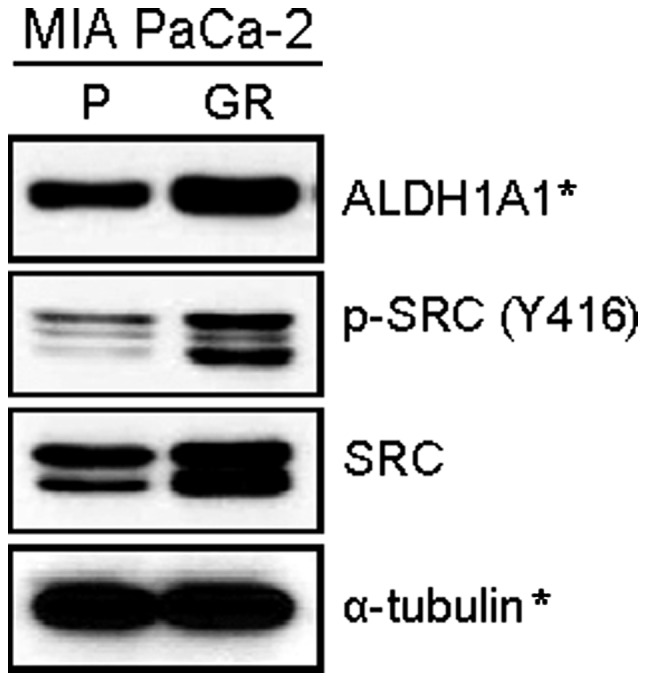
The level of phospho-SRC is higher in MIA PaCa-2/GR cells than in MIA PaCa-2/P cells. The western blot analysis of exponentially growing cells determined the level of the total and phosphorylated forms of SRC (Y416). α-tubulin was used for a loading and transfer control. ^*^Adopted from our previous report (20).

Our recent study showed that among various human pancreatic cancer cell lines, MIA PaCa-2 cells exhibit the highest level of ALDH1A1 (20), a marker of tumor-initiating and/or cancer stem cell properties in human pancreatic cancer. In addition, we also found that the level of ALDH1A1 expression was higher in MIA PaCa-2/GR cells than MIA PaCa-2/P cells (20). Given the potential correlation of SRC with ALDH1A1, we first examined the level of SRC in both MIA PaCa-2/P and MIA PaCa-2/GR cells. The western blot analysis revealed that both phosphorylated and total form of SRC was significantly higher in MIA PaCa-2/GR cells than MIA PaCa-2/P cells (Fig. 1).

### The synergistic antitumor effects by combination of dasatinib and gemcitabine

**Figure 2.**
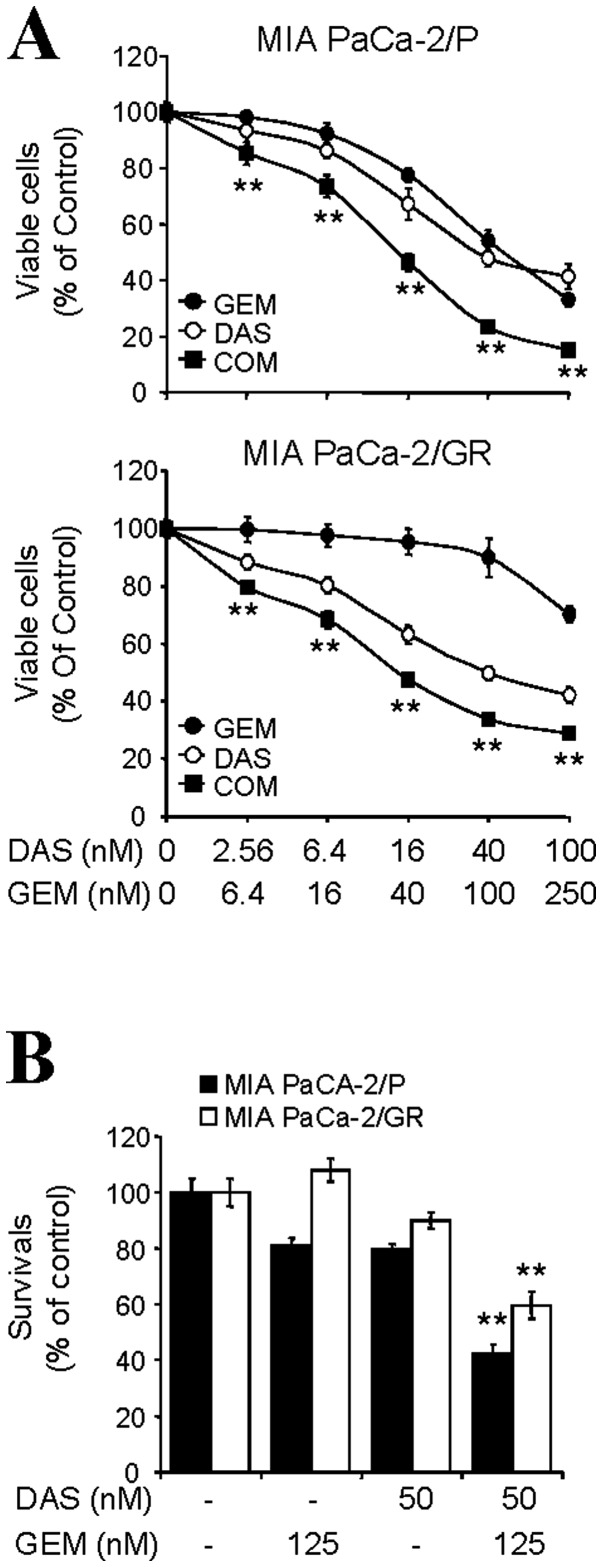
The synergistic anti-proliferative effects by combination of dasatinib (DAS) and gemcitabine (GEM). (A) An MTT assay was used to determine the cell viability of MIA PaCa-2/P and MIA PaCa-2/GR cells that co-treated with DAS and GEM with various concentrations for 72 h with fixed molar concentration ratios of 1:2.5. Data from two independent experiments performed in triplicate are shown as mean ± SD. (B) A clonogenic assay was performed to determine the long-term response of MIA PaCa-2/P and MIA PaCa-2/GR cells that treated with either DAS (50 nM), GEM (125 nM) alone or in combination of both drugs for 24 h. Colony numbers were counted and calculated as a relative percentage (%) of the untreated control cells. Experiments were repeated three times and similar results were obtained. Data are presented mean ± SD. ^**^P<0.01.

In order to investigate the potential beneficial effect of dasatinib/gemcitabine combination, both cell lines were treated with dasatinib and gemcitabine for 72 h with fixed molar ratios of 1:2.5. As shown in Fig. 2A, combination of dasatinib/gemcitabine synergistically reduced the viable cells in both cell lines. The CI values at ED_50_, ED_75_, and ED_90_ are 0.67, 0.74 and 0.86 for MIA PaCa-2/P cells and 0.47, 0.57 and 0.79 for MIA PaCa-2/GR cells, respectively (Table I). In order to determine the long-term combination effect of dasatinib/gemcitabine, cells were treated with either dasatinib, gemcitabine or in combination of both drugs for 24 h and then, cells were continually re-cultured in fresh media without drug for 14 days. Survived colonies were calculated as described in Materials and methods and plotted as percentages of the no treatment control. Results showed that either gemcitabine or dasatinib as single agent had limited effect on the survival of these cells. On the contrary, the cell survival was significantly reduced up to ∼42% in MIA PaCa-2/P and ∼59% in MIA PaCa-2/GR when the cells were co-treated with dasatinib/ gemcitabine (Fig. 2B). These results suggest that the combination of dasatinib/gemcitabine results in a synergistic decrease in the cell proliferation and survival of ALDH1A1-enriched pancreatic cancer MIA PaCa-2 cells.

### Dasatinib enhances apoptotic cell death by gemcitabine in MIA PaCa-2 cells

**Figure 3.**
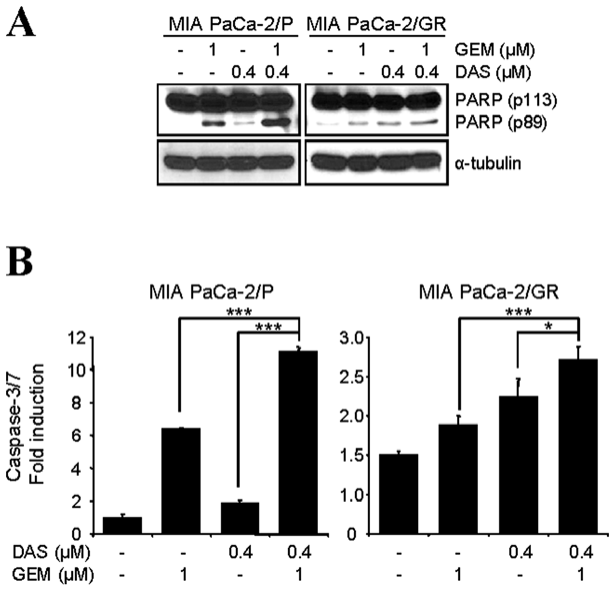
Dasatinib (DAS) enhances gemcitabine (GEM)-induced apoptotic cell death. (A) The cells were treated with either DAS (0.4 *μ*M), GEM (1 *μ*M) alone or in combination of both drugs for 48 h and then western blot analysis was performed with indicated antibodies. α-tubulin was used for a loading and transfer control. (B) The cells treated as described in (A) were lysed and caspase-3/7 activities were measured as described in Materials and methods. Data from three independent experiments are shown as mean ± SD. ^*^P<0.05 and ^***^P<0.005.

The combination of dasatinib/gemcitabine on the induction of apoptosis was also investigated. Cells were treated with either dasatinib (0.4 *μ*M), gemcitabine (1 *μ*M) alone or in combination of both drugs for 48 h and western blot analysis was performed to detect apoptotic cell death with a molecular biomarker of apoptosis, PARP cleavage. Comparing to cells treated with dasatinib (0.4 *μ*M) or gemcitabine (1 *μ*M) alone, dasatinib/gemcitabine-treated cells showed a more dramatic increase of PARP cleavage in both cell lines (Fig. 3A). As expected, the combination of dasatinib/gemcitabine significantly increased the caspase-3/7 activity in both cell lines (Fig. 3B). Taken together, these results suggest that treatment of dasatinib significantly enhances gemcitabine-induced apoptosis through activation of caspase-3/7 and PARP cleavage in both MIA PaCa-2/P and MIA PaCa-2/GR cell lines.

### Dasatinib/gemcitabine combination inhibits the level of ALDH1A1 expression and phospho-SRC

**Figure 4.**
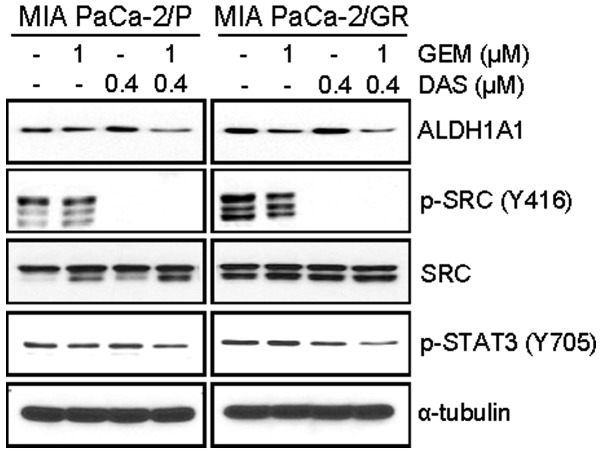
Dasatinib (DAS) and gemcitabine (GEM) combination reduces the level of ALDH1A1 expression and phospho-STAT3 (Y705) in both MIA PaCa-2/P and MIA PaCa-2/GR cells. The cells were treated as indicated for 48 h and western blot analysis was performed with indicated antibodies. α-tubulin was used for a loading and transfer control.

We also evaluated the effect of dasatinib/gemcitabine combination on ALDH1A1 expression. Treatment of cells for 48 h with gemcitabine (1 *μ*M) partly reduced the basal expression of ALDH1A1, while dasatinib (0.4 *μ*M) did not affect the basal expression of ALDH1A1 (Fig. 4). Importantly, the combination of dasatinib/ gemcitabine further reduced the level of ALDH1A1 expression in both cell lines (Fig. 4).

Since dasatinib has been reported to decrease phosphorylation of SRC, FAK, and AKT in a concentration-dependent manner in either gemcitabine-sensitive (BxPC3) or gemcitabine-resistant (Panc-1) pancreatic cancer cells (38), we also determined the level of phospho-SRC in dasatinib/gemcitabine-treated cells. The western blot analysis showed that combination of dasatinib/gemcitabine did not affect the dasatinib-mediated suppression of phospho-SRC (Y416) in these cells (Fig. 4). Under these conditions, dasatinib alone showed limited inhibitory effect on the phospho-STAT3 (Y705). However, combination of dasatinib/gemcitabine inhibited the phosphorylation of STAT3 (Y705) (Fig. 4).

### Knockdown of SRC increases apoptotic cell death and decreases the level of ALDH1A1 expression by gemcitabine

**Figure 5.**
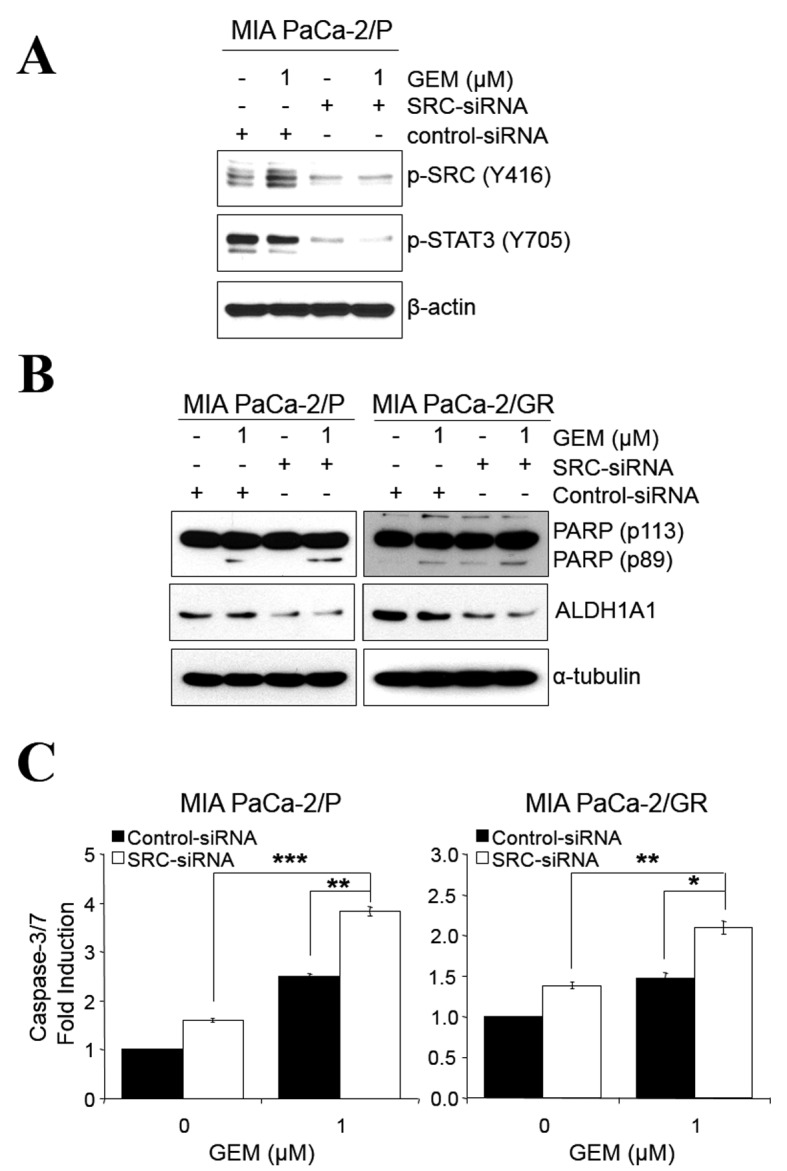
Knockdown of SRC enhances gemcitabine (GEM)-induced apoptotic cell death and reduces the level of ALDH1A1 expression. (A) MIA PaCa-2/P cells were transfected with either SRC-siRNA or control-siRNA for 48 h and further treated with GEM (1 *μ*M) for 48 h. The western blot analysis was conducted to determine the indicated proteins. β-actin was used for a loading and transfer control. (B) The cells were transfected with either SRC-siRNA or control-siRNA for 48 h and further treated with GEM (1 *μ*M) for 48 h and western blot analysis was performed with indicated antibodies. α-tubulin was used for a loading and transfer control. (C) The cells treated as described in (B) were lysed and the caspase-3/7 activities were determined as described in Materials and methods. Representative data are shown as mean ± SD from three independent experiments. ^*^P<0.05; ^**^P<0.01; and ^***^P<0.005.

The enhanced effect of SRC inhibition on gemcitabine-induced cell death was further confirmed by siRNA-mediated knockdown. The cells were transfected with either control- or SRC-siRNA and further treated with gemcitabine. Under these conditions, knockdown of SRC by siRNA reduced the level of phospho-SRC (Y416) and phospho-STAT3 (Y705). Consistent with dasatinib/gemcitabine combination, combination of SRC-siRNA and gemcitabine further suppressed the phospho-STAT3 (Y705) in MIA PaCa-2/P cells (Fig. 5A).

In order to confirm whether inhibition of SRC enhances sensitivity to gemcitabine, cells pretreated with either SRC-siRNA or control-siRNA for 48 h were incubated with various concentrations of gemcitabine for 72 h and cell viability was determined by MTT assay. The results showed that combination of SRC-siRNA and gemcitabine was significantly more effective in reducing the viable cells than control-siRNA and gemcitabine in both cell lines (data not shown). Furthermore, gemcitabine-induced PARP cleavage and caspase-3/7 activity were enhanced in these cells by knockdown of SRC (Fig. 5B and C). In addition, SRC-knockdown reduced the level of ALDH1A1 in both cell lines (Fig. 5B). Again, combination of SRC-siRNA and gemcitabine further decreased the level of ALDH1A1 expression (Fig. 5B). Taken together, targeting of SRC activity by dasatinib or siRNA enhanced gemcitabine-induced cell death by inducting apoptotic cell death and reducing the level of ALDH1A1 expression in ALDH1A1-enriched pancreatic cancer MIA PaCa-2 cells.

## Discussion

In this study, we investigated the effect of dasatinib on gemcitabine-sensitivity of ALDH1A1-enriched pancreatic cancer MIA PaCa-2 cells. As results, we found that: i) MIA PaCa-2/GR cells express significantly higher levels of phospho-SRC and total SRC as well as ALDH1A1 than MIA PaCa-2/P cells; ii) combination of dasatinib/gemcitabine reduces the cell proliferation and survival by induction of apoptotic cell death in both MIA PaCa-2/P and MIA PaCa-2/ GR cells; iii) dasatinib/gemcitabine combination inhibits the phosphorylation of SRC (Y416) and STAT3 (Y705) and reduces the level of ALDH1A1; iv) knockdown of SRC by siRNA enhances gemcitabine-induced apoptotic cell death; and v) combination of SRC-knockdown with gemcitabine reduced the level of ALDH1A1 expression. These results strongly support that inhibition of SRC by dasatinib or siRNA sensitizes ALDH1A1-enriched pancreatic cancer MIA PaCa-2 cells to gemcitabine.

Failure of standard chemotherapy and radiotherapy to eliminate pancreatic CSCs is thought to be a key hurdle in the treatment of pancreatic cancer. CSCs including pancreatic CSCs possess potential to self-renew and to differentiate into heterogeneous population of cells within the tumor, and have a high ability to invade and metastasize (39,40). It has been reported that pancreatic CSCs are resistant to gemcitabine and therefore play a significant role in recurrence of primary pancreatic cancer (41–43). Pancreatic CSCs are currently identified by the expression of specific surface and cytoplasmic markers. The potential stem cell markers such as CD24, CD44 and ESA were reported to be enriched in pancreatic CSCs (44); and other markers such as CD133 and ALDH1A1 were also reported to be enriched in pancreatic CSCs (16,44,45). Recently, ALDH1A1 has been identified as a potential therapeutic target (7,46). Notably, inhibition of ALDH1A1 has been shown to sensitize ALDH1A1-enriched pancreatic cancer MIA PaCa-2 cells to gemcitabine (20); in addition, inhibition of ALDH has been also shown to sensitize ALDH-enriched ovarian and breast cancer cells to chemotherapy and radiotherapy (18,47).

Taken together, the present study suggests a potential application of dasatinib/gemcitabine combination to eliminate the CSC population in the ALDH1A1-enriched pancreatic cancer MIA PaCa-2 cell lines. This might be particularly of importance in treatment of pancreatic CSCs which show resistance to gemcitabine (41–43). Our observation implicates that the combination of dasatinib/gemcitabine may provide great benefit to eliminate pancreatic CSCs and inhibit the recurrence of pancreatic cancer. However, further studies are needed to investigate this therapeutic strategy in ALDH1A1-positive cells that are isolated by Aldeflour assay or other CSC marker-specific methods both *in vitro* and *in vivo* animal models.

## Figures and Tables

**Table I.** Synergistic inhibition of cell proliferation by combination of dasatinib/gemcitabine in ALDH1A1-enriched pancreatic cancer MIA PaCa-2 cells.^a^

	ED_50_	ED_75_	ED_90_
MIA PaCa-2/P	0.67	0.74	0.86
MIA PaCa-2/GR	0.47	0.57	0.79

a These CI values were calculated as described in Materials and methods. Values of CI <1, =1 and >1 indicate synergism, additive effects and antagonism, respectively.

## References

[1] Hidalgo M (2010). Pancreatic cancer. N Engl J Med.

[2] Long J, Zhang Y, Yu X, Yang J, LeBrun DG, Chen C, Yao Q, Li M (2011). Overcoming drug resistance in pancreatic cancer. Expert Opin Ther Targets.

[3] Duffy JP, Eibl G, Reber HA, Hines OJ (2003). Influence of hypoxia and neoangiogenesis on the growth of pancreatic cancer. Mol Cancer.

[4] Conroy T, Desseigne F, Ychou M, Bouché O, Guimbaud R, Bécouarn Y, Adenis A, Raoul JL, Gourgou-Bourgade S, de la Fouchardière C, Bennouna J, Bachet JB, Khemissa-Akouz F, Péré-Vergé D, Delbaldo C, Assenat E (2011). FOLFINOX versus gemcitabine for metastatic pancreatic cancer. N Engl J Med.

[5] Loehrer PJ, Feng Y, Cardenes H, Wagner L, Brell JM, Cella D, Flynn P, Ramanathan RK, Crane CH, Alberts SR, Benson AB (2011). Gemcitabine alone versus gemcitabine plus radiotherapy in patients with locally advanced pancreatic cancer: an Eastern Cooperative Oncology Group trial. J Clin Oncol.

[6] Kim MP, Gallick GE (2008). Gemcitabine resistance in pancreatic cancer: picking the key players. Clin Cancer Res.

[7] Marcato P, Dean CA, Giacomantonio CA, Lee PWK (2011). Aldehyde dehydrogenase: its role as a cancer stem cell marker comes down to the speficic isoform. Cell Cycle.

[8] Dylla SJ, Beviglia L, Park IK, Chartier C, Raval J, Ngan L, Pickell K, Aguilar J, Lazetic S, Smith-Berdan S, Clarke MF, Hoey T, Lewicki J, Gurney AL (2008). Colorectal cancer stem cells are enriched in xenogeneic tumors following chemotherapy. PLoS One.

[9] Magni M, Shammah S, Schiró R, Mellado W, Dalla-Favera R, Gianni AM (1998). Induction of cyclophosphamide-resistance by aldehyde-dehydrogenase gene transfer. Blood.

[10] Sládek NE, Kollander R, Sreerama L, Kiang DT (2002). Cellular levels of aldehyde dehydrogeneases (ALDH1A1 and ALDH3A1) as predictors of therapeutic responses to cyclophosphamide-based chemotherapy of breast cancer: a retrospective study. Rational individualization of oxazaphosphorine-based cancer chemotherapeutic regimens. Cancer Chemother Pharmacol.

[11] Charafe-Jauffret E, Ginestier C, Iovino F, Tarpin C, Diebel M, Esterni B, Houvenaeghel G, Extra JM, Bertucci F, Jacquemier J, Xerri L, Dontu G, Stassi G, Xiao Y, Barsky SH, Birnbaum D (2010). Aldehyde dehydrogenase 1-positive cancer stem cells mediate metastasis and poor clinical outcome in inflammatory breast cancer. Clin Cancer Res.

[12] Huang EH, Hynes MJ, Zhang T, Ginestier C, Dontu G, Appelman H, Fields JZ, Wicha MS, Boman BM (2009). Aldehyde dehydrogenase 1 is a marker for normal and malignant human colonic stem cells (SC) and tracks SC overpopulation during colon tumorigenesis. Cancer Res.

[13] Su Y, Qui Q, Zhang Z, Jiang Z, Leng Q, Liu Z, Stass SA, Jiang F (2010). Aldehyde dehydrogenase 1 A1-positive cell population is enriched in tumor-initiating cells and associated with progression of bladder cancer. Cancer Epidemiol Biomarkers Prev.

[14] Dembinski JL, Krauss S (2009). Characterization and functional analysis of a slow cycling stem cell-like subpopulation in pancreas adenocarcinoma. Clin Exp Metastasis.

[15] Clay MR, Tabor M, Owen JH, Carey TE, Bradford CR, Wolf GT, Wicha MS, Prince ME (2010). Single-marker identification of head and neck squamous cell carcinoma cancer stem cells with aldehyde dehydrogenase. Head Neck.

[16] Jiang F, Qui Q, Khanna A, Todd NW, Deepak J, Xing L, Wang H, Liu Z, Su Y, Stass SA, Katz RL (2009). Aldehyde dehydrogenase 1 is a tumor stem cell-associated marker in lung cancer. Mol Cancer Res.

[17] Ma S, Chan KW, Hu L, Lee TK, Wo JY, Ng IO, Zheng BJ, Guan XY (2007). Identification and characterization of tumorigenic live cancer stem/progenitor cells. Gastroenterology.

[18] Landen CN, Goodman B, Katre AA, Steg AD, Nick AM, Stone RL, Miller LD, Mejia PV, Jennings NB, Gershenson DM, Bast RC, Coleman RL, Lopez-Berestein G, Sood AK (2010). Targeting aldehyde dehydrogenase cancer stem cells in ovarian cancer. Mol Cancer Ther.

[19] Rasheed ZA, Yang J, Wang Q, Kowalski J, Freed I, Murter C, Hong SM, Koorstra JB, Rajeshkumar NV, He X, Goggins M, Iacobuzio-Donahue C, Berman DM, Laheru D, Jimeno A, Hidalgo M, Maitra A, Matsui W (2010). Prognostic significance of tumorigenic cells with mesenchymal features in pancreatic adenocarcinoma. J Natl Cancer Inst.

[20] Duong HQ, Hwang JS, Kim HJ, Kang HJ, Seong YB, Bae I (2012). Aldehyde dehydrogenase 1A1 confers intrinsic and acquired resistance to gemcitabine in human pancreatic adenocarcinoma MIA PaCa-2 cells. Int J Oncol.

[21] Araujo J, Logothetis C (2010). Dasatinib: a potent SRC inhibitor in clinical development for the treatment of solid tumors. Cancer Treat Rev.

[22] Kim LC, Rix U, Haura EB (2010). Dasatinib in solid tomors. Expert Opin Investig Drugs.

[23] Ceppi P, Papotti M, Monica V, Lolanono M, Saviozzi S, Pautasso M, Novello S, Mussino S, Bracco E, Volante M, Scagliotti GV (2009). Effects of Src kinase inhibition induced by dasatinib in non-small cell lung cancer cell lines treated with cisplatin. Mol Cancer Ther.

[24] Kopetz S, Lesslie DP, Dallas NA, Park SI, Johnson M, Parikh NU, Kim MP, Abbruzzese JL, Ellis LM, Chandra J, Gallick GE (2009). Synergistic activity of the SRC family kinase inhibitor dasatinib and oxaliplatin in colon carcinoma cells is mediated by oxidative stress. Cancer Res.

[25] Nagaraj NS, Washington MK, Merchant NB (2011). Combined blockade of Src kinase and epidermal growth factor receptor with gemcitabine overcomes STAT3-mediated resistance of inhibition of pancreatic tumor growth. Clin Cancer Res.

[26] Kim YJ, Hong YB, Cho CH, Seong YS, Bae I (2012). Exploring protein kinase inhibitors: unveiling gemcitabine resistance in pancreatic cancer. Pancreas.

[27] Hong DS, Choe JH, Naing A, Wheler JJ, Falchook GS, Piha-Paul S, Moulder SL, George GC, Choe JM, Strauss LC, Gallick GE, Kurzrock R (2013). A phase 1 study of gemcitabine combinated with dasatinib in patients with advanced solid tumors. Invest New Drugs.

[28] Park BJ, Whichard ZL, Corey SJ (2012). Dasatinib synergizes with both cytotoxic and signal transduction inhibitors in heterogeneous breast cancer cell lines - lessons for design of combination targeted therapy. Cancer Lett.

[29] Somlo G, Atzori F, Strauss LC, Geese WJ, Specht JM, Gradishar WJ, Rybicki A, Sy O, Vahdal LT, Cortes J (2013). Dasatinib plus capecitabine for advanced breast cancer: safety and efficacy in phase I study CA180004. Clin Cancer Res.

[30] Araujo JC, Mathew P, Armstrong AJ, Braud EL, Posadas E, Lonberg M, Gallick GE, Trudel GC, Paliwal P, Agrawal S, Logothetic CJ (2012). Dasatinib combined with docetaxel for castration-resistant prostate cancer: results from a phase 1–2 study. Cancer.

[31] Secord AA, Teoh DK, Barry WT, Yu M, Broadwater G, Havrilesky LJ, lee PS, Berchuck A, Lancaster J, Wenham RM (2012). A phase I trial of dasatinib, an SRC-family kinase inhibitor, in combination with paclitaxel and carboplatin in patients with advanced or recurrent ovarian cancer. Clin Cancer Res.

[32] Algazi AP, Weber JS, Andrews SC, Urbas P, Munster PN, DeConti RC, Hwang J, Sondak VK, Messina JL, McCalmont T, Daud AI (2012). Phase I clinical trial of the Src inhibitor dasatinib with dacarbazine in metastatic melanoma. Br J Cancer.

[33] Breccia M, Serrao A, Salaroli A, Loglisci G, Zacheo I, Alimena G (2012). Dasatinib combined with weekly administration of vincristine as effective therapy in sudden or resistant Ph+ lymphoid blast crisis of chronic myeloid leukaemia. Br J Haematol.

[34] Kurebayashi J, Kanomata N, Moriya T, Kozuka Y, Watanabe M, Sonoo H (2010). Preferential antitumor effect of the Src inhibitor dasatinib associated with a decreased proportion of aldehyde dehydrogenase 1-positive cells in breast cancer cells of the basal B subtype. BMC Cancer.

[35] Nautiyal J, Kanwar SS, Yu Y, Majumdar AP (2011). Combination of dasatinib and curcumin eliminates chemo-resistant colon cancer cells. J Mol Signal.

[36] Duong HQ, Kim HJ, Kang HJ, Seong YS, Bae I (2012). ZSTK474, a PI3K inhibitor, suppresses proliferation and sensitizes human pancreatic adenocarcinoma cells to gemcitabine. Oncol Rep.

[37] Duong HQ, Hong YB, Kim JS, Lee HS, Yi YW, Kim YJ, Wang A, Zhao W, Cho CH, Seong YS, Bae I (2013). Inhibition of checkpoint kinase 2 (CHK2) enhances sensitivity of pancreatic adenocarcinoma cells to gemcitabine. J Cell Mol Med.

[38] Nagaraj NS, Smith JJ, Revetta F, Washington MK, Merchant NB (2010). Targeted inhibition of SRC kinase signaling attenuates pancreatic tumorigenesis. Mol Cancer Ther.

[39] Dick JE (2008). Stem cell concepts renew cancer research. Blood.

[40] Wang JC, Dick JE (2005). Cancer stem cells: lesions from leukemia. Trends Cell Biol.

[41] Yin T, Wei H, Gou S, Shi P, Yang Z, Zhao G, Wang C (2011). Cancer stem-like cells enriched in panc-1 spheres possess increased migration ability and resistance to gemcitabine. Int J Mol Sci.

[42] Hu G, Li F, Ouyang K, Xie F, Tang X, Wang K, Han S, Jiang Z, Zhu M, Wen D, Qin X, Zhang L (2012). Intrinsic gemcitabine resistance in a novel pancreatic cancer cell line is associated with cancer stem cell-like phenotype. Int J Oncol.

[43] Hage C, Rausch V, Giese N, Giese T, Schonsiegel F, Labsch S, Nwaeburu C, Mattern J, Gladkich J, Herr I (2013). The novel c-Met inhibitor cabozantinib overcomes gemcitabine resistance and stem cell signaling in pancreatic cancer. Cell Death Dis.

[44] Li C, Heidt DG, Dalerba P, Burant CF, Zhang L, Adsay V, Wicha M, Clarke MF, Simeone DM (2007). Identification of pancreatic cancer stem cells. Cancer Res.

[45] Kim MP, Fleming JB, Wang H, Abbruzzese JL, Choi W, Kopetz S, McConkey DJ, Evans DB, Gallick GE (2011). ALDH activity selectively defines an enhanced tumor-initiating cell population relative to CD133 expression in human pancreatic adenocarcinoma. PLoS One.

[46] Koppaka V, Thompson DC, Chen Y, Ellermann M, Nicolaou KC, Juvonen RO, Petersen D, Deitrich RA, Hurley TD, Vasiliou V (2012). Aldehyde dehydrogenase inhibitors: a comprehensive review on the pharmacology, mechanism of action, substrate specificity, and clinical application. Pharmacol Rev.

[47] Croker AK, Allan AL (2012). Inhibition of aldehyde dehydrogenase (ALDH) activity reduces chemotherapy and radiation resistance of stem-like ALDHhiCD44^+^ human breast cancer cells. Breast Cancer Res Treat.

